# From General Aberrant Alternative Splicing in Cancers and Its Therapeutic Application to the Discovery of an Oncogenic DMTF1 Isoform

**DOI:** 10.3390/ijms18030191

**Published:** 2017-03-02

**Authors:** Na Tian, Jialiang Li, Jinming Shi, Guangchao Sui

**Affiliations:** 1 College of Life Science, Northeast Forestry University, Harbin 150040, China; tianna@nefu.edu.cn (N.T.); lijialiang@nefu.edu.cn (J.L.); jmshi@nefu.edu.cn (J.S.); 2 Department of Cancer Biology and Comprehensive Cancer Center, Wake Forest University School of Medicine, Winston-Salem, NC 27157, USA

**Keywords:** alternative splicing, *DMTF1*, tumorigenesis, cancer therapy

## Abstract

Alternative pre-mRNA splicing is a crucial process that allows the generation of diversified RNA and protein products from a multi-exon gene. In tumor cells, this mechanism can facilitate cancer development and progression through both creating oncogenic isoforms and reducing the expression of normal or controllable protein species. We recently demonstrated that an alternative cyclin D-binding myb-like transcription factor 1 (DMTF1) pre-mRNA splicing isoform, DMTF1β, is increasingly expressed in breast cancer and promotes mammary tumorigenesis in a transgenic mouse model. Aberrant pre-mRNA splicing is a typical event occurring for many cancer-related functional proteins. In this review, we introduce general aberrant pre-mRNA splicing in cancers and discuss its therapeutic application using our recent discovery of the oncogenic DMTF1 isoform as an example. We also summarize new insights in designing novel targeting strategies of cancer therapies based on the understanding of deregulated pre-mRNA splicing mechanisms.

## 1. Introduction

Pre-mRNA splicing is a key step for the maturation of transcripts of multi-exon genes in eukaryotes. It allows one genomic coding locus to encode multiple functionally distinct isoforms of noncoding RNAs (ncRNAs) or proteins and thus extends the capacity of eukaryotic genomes [1]. As an example, the gene locus of *DMTF1* (cyclin D-binding myb-like transcription factor 1), also known as *DMP1* (cyclin D-binding myb-like protein 1), encodes three major isoforms with different functions in cancers [2,3]. In the human genome, about 95% of exon-containing genes undergo alternative splicing, which plays a major role in generating the high diversity of cellular transcripts and proteins [4]. The products of these alternatively spliced RNA, both ncRNAs and translated proteins, also contribute to the functional diversity of regulatory molecules in various signaling pathways and biological processes involving in cell proliferation, differentiation, immortalization, apoptosis, etc. Deregulated pre-mRNA splicing process results in aberrant RNA variants, significantly impacting on many human diseases, including cancers [5].

Most cancers are heterogeneous at the genomic and histological levels. At the genomic level, cancers consist of cells with different genetic and epigenetic alterations [6]. At the cellular level, overexpressed oncogenes or mutated tumor suppressors drive deregulated signaling pathways or cascades to promote cancer development and progression. In addition to the genetic and epigenetic alterations, other mechanisms can also contribute to tumorigenesis. Aberrant alternative RNA splicing produces ncRNA or protein molecules with distinct or opposite functions against its regular cognate products and consequently contributes to malignant transformation. Dysregulated pre-mRNA splicing in many cancer-related genes, such as *TP53*, *MDM2*, and *BCL2L1*, contributes to cell proliferation, survival, genomic instability, and immortalization [5].

*DMTF1* is recognized as a *RAS*/*ERBB2*-activated haplo-insufficient tumor suppressor [7]. Its apparent tumor suppressive role has been linked to its regulation of the *CDKN2A-TP53*, *MDM2-TP53*, *EBRR2*, *RAS-RAF*, and *CCND1* signaling pathways. Alternative splicing of DMTF1 pre-mRNA leads to the production of three isoforms, α, β, and γ [8]. We and others demonstrated the distinct oncogenic function of DMTF1β from DMTF1α in tumorigenesis [2,3,9]. The presence of different isoforms of DMTF1, as well as other cancer-related regulators, provides insights about new vulnerable targets in cancer therapies. In this review, we will first make a concise summary of alternative RNA splicing regulatory mechanisms, with a focus on pre-mRNAs of protein-coding genes, and its relevance to tumorigenesis. We will then introduce the splicing events and functional role of DMTF1 isoforms. We will use it as an example to discuss how alternative splicing may affect cancer-related signaling pathways and how the understanding of aberrant splicing can help us in designing approaches for cancer therapies.

## 2. Alternative Splicing: Mechanisms and Their Relevance to Cancers

### 2.1. General Mechanism of Pre-mRNA Splicing

Pre-mRNA splicing is a process to remove an intron sequence between two neighbor exons and then re-ligate the exons. Inside an intron, the 5′ end is the donor site, also called 5′ splice site, and usually contains a sequence GU; the 3′ end is the acceptor site, or 3′ splice site, and consists of a sequence of AG. The pre-mRNA splicing process consists of two-step transesterification reactions. First, the 2′ OH of a specific nucleotide in an intron (i.e., branch point, usually an adenosine close to the 3′ splice site) initiates a nucleophilic attack to the 5′ splice site. This leads to the formation of a lariat structure with a 2′,5′-phosphodiester linkage. Second, the 3′ OH at the free end of the upstream exon starts another nucleophilic attack to the first nucleotide of the downstream exon (i.e., the nucleotide right after the 3′ splice site). This results in the release of the intron lariat and re-ligation of the two exons [10].

**Figure 1:**
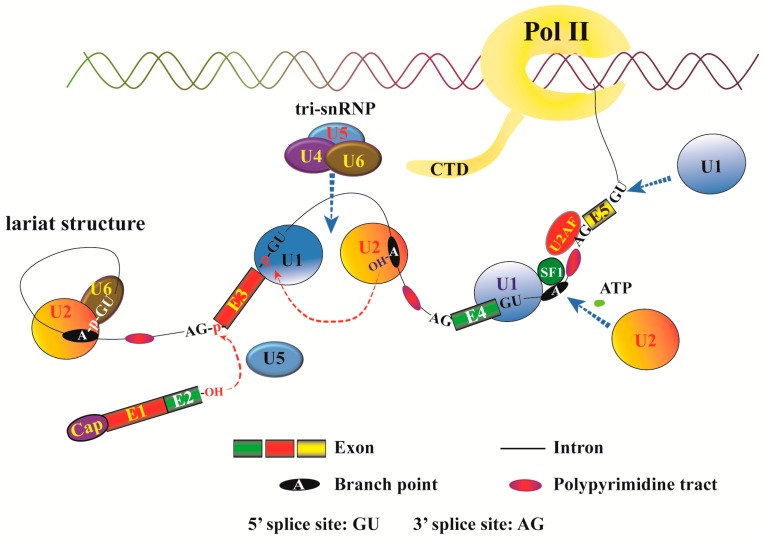
Schematic diagram of spliceosome assembly during pre-mRNA splicing. RNA transcription and pre-mRNA splicing can concurrently occur [13]. The representation depicts pre-mRNA splicing events among nascent exons 2, 3, 4, and 5 with already processed splicing between exons 1 and 2. A canonical spliceosome contains five small snRNPs, U1, U2, U4, U5, and U6. The 5′ and 3′ splice sites, branch points, and polypyrimidine tracts of the three introns are indicated. U1 snRNP, and splicing factors SF1 (splicing factor 1) and U2AF (U2 small nuclear RNA auxiliary factor) bind to the 5′ splice sites, branch points, and polypyrimidine tracts, respectively. Then, U2 snRNP replaces SF1 at the branch points. With the recruitment of the tri-snRNP consisting of U4, U5, and U6, the spliceosome assembly is completed [4]. “CTD” denotes the C-terminal domain of RNA Polymerase II (Pol II), which can be attached by a spliceosome [14]. Dotted blue arrows indicate protein binding or recruitment, while dotted red arrow lines show nucleophilic attacks.

Pre-mRNA splicing process is catalyzed by spliceosome, which can be categorized into the major and minor spliceosomes. The major spliceosome contains five small nuclear ribonucleoproteins (snRNPs), U1, U2, U4, U5, and U6 (Figure 1), and processes canonical splicing for over 95% of introns. The minor spliceosome consists of snRNPs U11, U12, U4atac, and U6atac, and catalyzes non-canonical intron splicing with splice site sequences different from these of the major spliceosome. Spliceosome recognition at the branch point, 5′ and 3′ splice sites is crucial to the splicing process. The exons and introns have short and degenerate elements named *cis*-acting exonic and intronic splicing enhancers (ESEs and ISEs, respectively), and exonic and intronic splicing silencers (ESSs and ISSs, respectively). These are the binding sites for different RNA-binding proteins [11]. A polypyrimidine tract of 15–20 nucleotides that is rich with pyrimidine nucleotides, especially uridine, is present at 5–40 nucleotides upstream of the 3′ splice site. Its function is promoting spliceosome assembly [12].

### 2.2. RNA-Binding Proteins and Their Aberrant Regulation in Cancers

Pre-mRNA splicing process is regulated by many RNA-binding proteins (RBPs) that determine the splice sites in pre-mRNAs [15,16]. Two common RBP families, serine/arginine-rich (SR) proteins and heterogeneous nuclear ribonucleoproteins (hnRNPs), have been well-characterized for their regulatory activities in pre-mRNA splicing. SR proteins are important for both constitutive pre-mRNA splicing and alternative splicing. Especially, they regulate exon inclusion through binding to the ESEs and ISE [16]. Meanwhile, SR proteins are involved in other biological processes, including transcription, mRNA nuclear export, translation, and nonsense-mediated decay (NMD) [17,18,19]. hnRNPs may cause exon skipping through their association with ESSs and ISSs.

RBPs are crucial to maintain correctly processed pre-mRNA splicing and determine ratios of final splicing products from a specific gene locus; thus, their unbalanced expression or activity can lead to production of deregulated transcript isoforms in different diseases, including cancers [15]. Recent studies revealed a variety of spliceosome-related mutations discovered in over half of patients suffering from myelodysplastic syndromes (MDS), suggesting a new leukemogenic pathway involving aberrant pre-mRNA splicing [20]. To date, the molecular mechanisms underlying the regulation of pre-mRNA splicing process has greatly advanced and many RBP members have been characterized for their roles in promoting the production of regular RNA transcripts and oncogenic isoforms in tumor cells [16].

#### 2.2.1. Serine/Arginine-Rich (SR) Proteins and Their Deregulation in Cancers

The basic structural composition for each member of the SR protein family consists of a RNA recognition motif (RRM) and arginine/serine-rich (RS) motif. Some of them may also have a RNA recognition motif homology, also recognized as atypical RRM. Serine/arginine splicing factor 1 (SRSF1, or ASF/SF2) is a well-characterized SR protein regulating both pre-mRNA splicing and other related processes, such as nuclear exporting of mature RNA and NMD [16]. The *SRSF1* gene itself is deregulated in various malignancies and is recognized as a proto-oncogene in human cancers [21]. Although most SR proteins stimulate exon inclusion during splicing, SRSF1 can promote a similar number of exon inclusion and skipping changes, implicating its role as either an activator or a repressor of splicing [22]. SRSF1 regulates alternative pre-mRNA splicing of a number of genes that are involved in tumorigenesis. For example, BIN1, as a tumor suppressor, interacts with MYC (v-myc avian myelocytomatosis viral oncogene homolog) and inhibits its proliferative activity [23]. Overexpressed *SRSF1* promotes the inclusion of BIN1 exon 12a, generating an isoform that lacks binding ability to MYC [24]. Similarly, SRSF1 contributes to the aberrant pre-mRNA splicing of pro-apoptotic gene *BIM* and impairs BIM-mediated apoptosis [25]. In response to DNA damage, SRSF1 also negatively regulates alternative splicing of MDM2 pre-mRNA that generates the MDM2-ALT1 isoform with tumorigenic properties [26]. Other reported genes with SRSF1-regulated alternative pre-mRNA splicing include *RPS6KB1*, *MKNK2*, and *CASP9* (also named *caspase 9*) [27,28,29].

Most of the other members of the SR family, including proteins SRSF2–12, have been demonstrated to regulate alternative pre-mRNA splicing of genes with various biological functions. The deregulation of some these proteins, such as SRSF2, SRSF3, SRSF5, and SRSF6, has been linked to alterations of many cancer-related processes, including cell growth and proliferation, apoptosis, senescence, and genomic stability [16,30].

#### 2.2.2. hnRNPs and Their Deregulation in Cancers

The hnRNP family consists of over a dozen members designated by particular letters. The RNA binding domains among these hnRNPs show high variation [16]. While most hnRNPs utilize a conserved RRM for RNA binding, some of them contain an atypical RRM and a couple of them have a K Homology (KH) domain that is responsible for both RNA binding and recognition [31]. In cancer cells, many hnRNPs are aberrantly expressed and thus contribute to tumorigenesis. Their dysregulation may alter various cancer-related processes, including oncogenic isoform production, DNA repair, genome stability and tumor cell metastasis. Consistently, promoter analyses demonstrated that the expression of *HNRNPA1*, *A2*, *D*, *F*, *H*, and *K* genes is regulated by oncogene products, such as E2F1, JUN, and MYC. The essential roles of some hnRNPs in cancer development and progression have been demonstrated in many reports [32,33,34,35,36,37]. For instance, as a multi-functional splicing factor, HNRNPL (heterogeneous nuclear ribonucleoprotein L) is overexpressed in oral squamous cell carcinoma and promotes expression of the full-length oncogenic SRSF3 protein. With reduced HNRNPL levels, the SRSF3 pre-mRNA can undergo an alternative splicing to include exon 4 that contains an in-frame stop codon leading to NMD or truncated protein [32]. Recently, Gautrey et al. demonstrated that HNRNPH1 regulates alternative splicing of ERBB2 (erb-b2 receptor tyrosine kinase 2, also known as HER2) pre-mRNA and its expression negatively correlates with an oncogenic HER2 variant [35].

SR proteins bind to ESE elements to promote exon use, while hnRNPs associates with ESS elements and block exon recognition; thus, proteins from these two families may antagonize each other. For instance, SRSF1 binding to the ESE element in exon 3 of HIV-1 TAT pre-mRNA prevents the association of HNRNPA1 to the same exon [38]. Similarly, HNRNPA1 can also antagonize the alternative splicing function of SRSF1 [39,40].

### 2.3. Different Patterns of Alternative Pre-mRNA Splicing in Cancers

**Figure 2:**
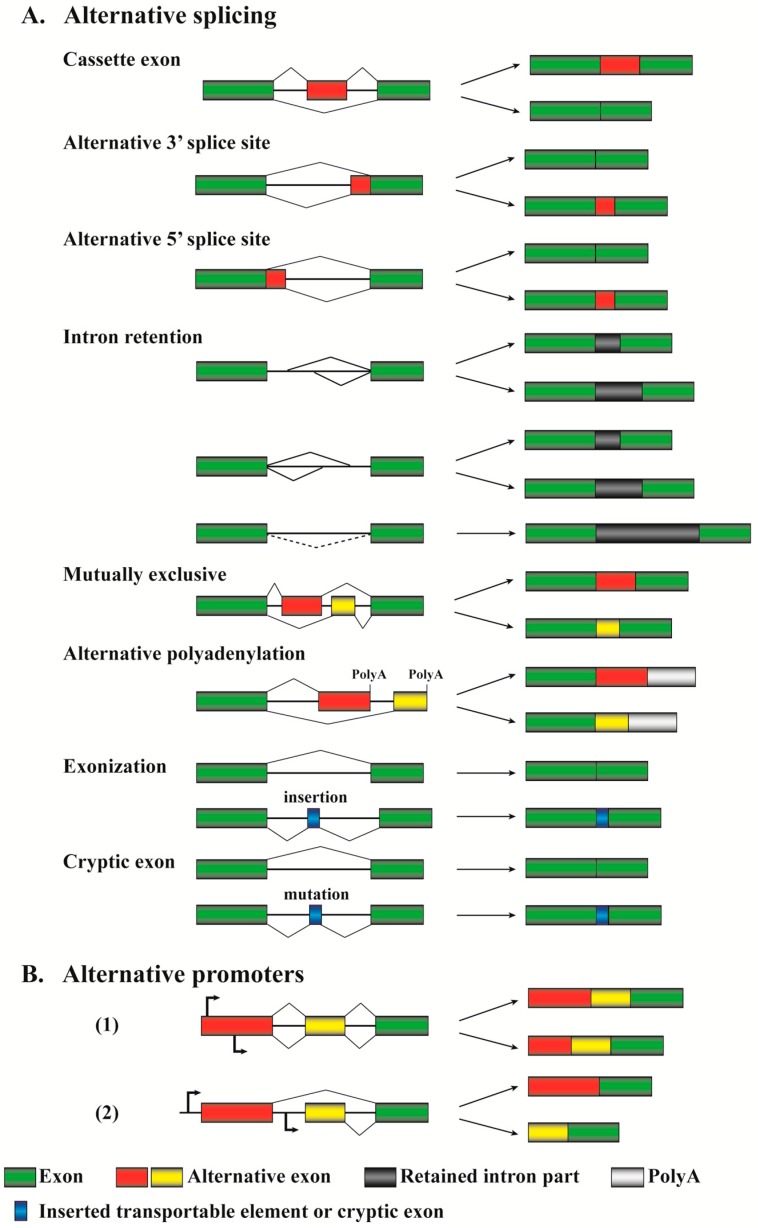
Schematic diagrams of the alternative splicing and alternative promoter patterns. (**A**) Alternative splicing. Exons and final transcripts are illustrated as boxes, while introns are represented by lines. Constitutively expressed exons are depicted in green, and alternatively spliced exons are in red or yellow. Folded lines are used to connect spliced ends. In the intron retention pattern, the intervening intron parts in the final transcripts are indicated by black boxes, while the dotted line represents no alternative splicing. PolyA sequences are depicted by grey boxes. In exonization and cryptic exon mechanisms, new exons (blue box) are generated by transportable element insertion or intronic sequence mutation; (**B**) Alternative promoters. The same representations are used as in “A”. Promoters are indicated by bent arrows. The upper arrows are the promoters for the transcription and pre-mRNA splicing indicated on the top, while the lower arrows indicate the promoters for the transcription and splicing at the bottom.

The availability of complete genomic sequences and data from RNA sequencing (RNA-seq) studies allow us to identify many novel alternative splicing variants of gene transcripts, most of which have unknown function and deserve further investigation [41]. Currently recognized alternative pre-mRNA splicing patterns are summarized in Figure 2A.

**Figure 3:**
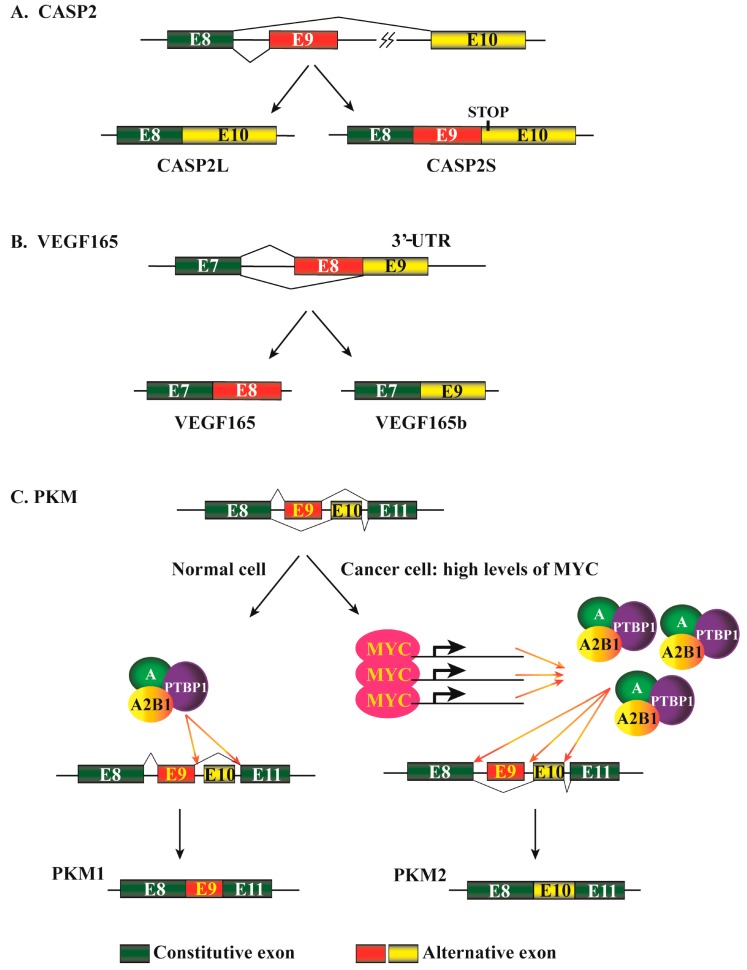
Alternative pre-mRNA splicing events of representative genes. (**A**) Cassette exons mechanism of alternative CASP2 pre-mRNA splicing. Constitutively expressed exons are depicted in green boxes, and alternatively spliced exons are in red or yellow boxes. Introns are represented by black lines with an omitted region indicated by a lightening sign. (These are the same in “B” and “C” below). The skipping of exon 9 leads to the formation of a CASP2 mRNA encoding a long version protein, CASP2L. Alternatively, exon 9 can be included in the mature mRNA that encodes a short version of the protein, CASP2S, due to the presence of a premature termination codon in exon 10; (**B**) Alternative 3′ splice site mechanism of alternative VEGF165 pre-mRNA splicing. In this mechanism, the 3′ end of exon 7 can be alternatively ligated to different sites of the 3′-UTR to form transcripts VEGF165 and VEGF165b, encoding proteins with distinct C-terminals; (**C**) Mutually exclusive mechanism of alternative PKM pre-mRNA splicing. In normal cells, exon 9 is typically retained while exon 10 is excluded. In cancer cells, the highly expressed MYC protein enhances the expression of *HNRNPA*, *HNRNPA2B1*, and *PTBP1* genes, which in turn promote an alternative splicing with exon 9 exclusion and exon 10 retention. The hnRNPs are represented by “A” for HNRNPA and “A2B1” for HNRNPA2B1. CASP2, caspase 2; PTBP1, polypyrimidine tract binding protein 1; MYC, v-myc avian myelocytomatosis viral oncogene homolog; VEGF165, vascular endothelial growth factor A 165; PKM, pyruvate kinase, muscle.

Cassette exons (or “exon inclusion or skipping”) are the most common events for regulating gene expression in both human and murine cells, and over 38% alternative splicing events are based on this mechanism [11]. This alternative splicing pattern allows excision of an entire exon(s) and its flanking introns from a pre-mRNA. Between exon inclusion and skipping for a particular gene, which mechanism can generate a transcript encoding a relative large or small protein depends on the reading frames of the two transcripts. Although the exon inclusion mechanism produces a longer transcript, the included exon may bring in a termination codon or shift the reading frame to create an earlier termination codon in downstream exon(s). This will lead to the production of a short version of the protein. Similarly, the exon skipping mechanism definitely generates a shorter transcript, but whether it encodes a relatively large or small protein relies on reading frame alteration. The considerations are applicable to other splicing mechanisms discussed below. *CASP2* (also named *caspase 2*) is one of the initiator caspases in apoptosis pathways. The skipping of its exon 9 during pre-mRNA splicing, promoted by SRSF3, leads to the formation of a long version of the protein, CASP2L, that induces apoptosis; when exon 9 is included, the generated splicing isoform contains a premature stop codon in exon 10 due to a reading frame shift and thus produces a short or truncated version of the protein (Figure 3A). CASP2S acts as an endogenous inhibitor of caspase activation and cell death [42,43,44].

The alternative 3′ splice site, or alternative acceptor site, represents about 18% of alternative splicing events [11]. This mechanism allows the same splicing donor site at a 5′ splice site to connect to alternative 3′ acceptor sites and thus generates products with different 5′ boundaries of the downstream exon. Vascular endothelial growth factor A (*VEGFA*) 165 (also named VEGF165) is a member of the PGF/VEGF growth factor family. It is a potent factor promoting angiogenesis and stimulating cell proliferation and migration. VEGF165b is generated by differential splicing from the 3′ end of exon 7 into different sites in the 3′ untranslated region of the mRNA (Figure 3B). The ectopic expression of VEGF165b inhibits VEGF165-mediated proliferation, migration of endothelial cells, and vasodilatation of mesenteric arteries [45].

The alternative 5′ splice, or alternative donor site, represents about 8% of slicing events [11]. It allows alternative 5′ splicing donor sites to connect to the same 3′ acceptor site and thus generates products with different 3′ boundaries of the upstream exon. Through this mechanism, the pre-mRNA of the *BCL2L1* (also named *Bcl-X*) gene can produce two isoforms, Bcl-XL and Bcl-XS, with opposite activities [46]. This will be further discussed below.

The intron retention pattern represents about 3% of alternative splicing events [11]. In general, intron retention is considered a rare pattern in mammals; however, it is a widespread mechanism for tumor suppressor inactivation in cancers [47]. This alternative splicing mechanism allows a part(s) or an entire intron to be included in the mature mRNA (Figure 2A). The generation of three DMTF1 isoforms utilizes this mechanism with partial retention of intron 9, which will be comprehensively discussed below. Other examples include *TP53*, *CDH1*, and *MLL3*, which mostly form truncated inactive mutants through the intron retention mechanism [47]. It is worthwhile to discuss the difference between alternative 3′ or 5′ splice sites and intron retention due to their apparent similarity. Their distinction is based on the definition of exon or intron lengths. For instance, in the mechanisms of alternative 3′ or 5′ splice sites, the red–green region (Figure 2A) can be generally recognized as whole exons and the green region alone is a partial exon. In contrast, in the mechanisms of intron retention, the lines between two green regions (exons) are generally taken as introns.

In addition to the splice patterns discussed above, other mechanisms, most of which are very sophisticated, represent about 33% of the total alternative pre-mRNA splicing events [11]. A mutually exclusive pattern allows one of two consecutive exons, but not both, to be included in the mature mRNA (Figure 2A). This mechanism involves two or more splicing events that are executed or disabled in a coordinated manner [48]. The pyruvate kinase muscle (*PKM*) gene is involved in cellular energy regeneration through producing ATP and pyruvate. During PKM pre-mRNA maturation, exons 9 and 10 are alternatively retained in a mutually exclusive manner, producing PKM1 and PKM2 isoforms (Figure 3C). In cancer cells, PKM2 is favorably expressed through mechanisms mediated by MYC, HNRNPA, HNRNPA2B1, and PTBP1 [49,50].

Another alternative splicing mechanism is alternative polyadenylation. In eukaryotes, pre-mRNA polyadenylation is one of the 3′ end modifications essential for mRNA maturation. A large portion of eukaryotic genes have pre-mRNAs with multiple alternative 3′ ends to be cleaved and polyadenylated at distinct sites, a phenomenon recognized as alternative polyadenylation [51]. Many oncogenes can be activated by alternative cleavage and polyadenylation at their 3′-untranslated region (UTR) in cancer cells, such as *CCND1* and *IGF2BP1* [52].

Exonization is defined as recruitment of a new exon from non-protein-coding, intronic DNA sequences. Some transposable elements, such as *Alu* sequences, can be inserted into intronic regions of genes and may generate new splicing sites to initiate exonization (Figure 2A) [53]. These events can enhance the diversity of cellular RNA and protein products, and contribute to transcriptome evolution [54]. To maintain genomic stability, eukaryotic cells have developed defense mechanisms to reduce the integration of transposable elements. Zarnack et al. reported that HNRNPC competed with the splicing factor U2AF2 to protect the human transcriptome from aberrant exonization of transposable elements [55]. Related to exonization, a cryptic exon is a rare alternative pre-mRNA splicing mechanism and represents the inclusion of a part of an intron into a mature mRNA. This mechanism can be initiated by mutations in an intron that may generate a strong splice site (Figure 2A). The *BRCA2* gene contains 27 exons and its encoded protein acts as a tumor suppressor through maintaining genomic stability [56]. A familial “T to G” mutation in intron 12 of the *BRCA2* gene reinforces the strength of a preexisting 5′ splice site. This results in the inclusion of a cryptic exon in intron 12 of the mature BRCA2 mRNA, leading to an insertion of a 95-nucleotide sequence between exons 12 and 13 [57].

Eukaryotes can also use a mechanism known as alternative promoters to produce different proteins from a single genomic locus (Figure 2B). Although transcribing RNA variants from the same locus, they are actually different genes driven by distinct promoters. The transcripts may have extensive overlapped regions and unique sequences for each, but the encoded proteins may not have any similarity due to reading frame shift. A classic example for the alternative promoter mechanism in cancers is one gene locus (*CDKN2A*) in the chromosome 9p21 encoding for two tumor suppressors, p14ARF and p16INK4A, which positively regulates *TP53* and *RB1*, respectively [58]. Although the transcripts of these two genes are mistakenly taken as two alternatively spliced isoforms in a number of reports, they are just partially overlapped mRNAs transcribed by two different promoters. This locus is frequently mutated, deleted, or epigenetically silenced in cancers. The consequent inactivation of p14ARF and p16INK4A causes large impacts leading to malignant transformation or cancer progression [59].

Aberrant pre-mRNA splicing is very common in cancer cells [5]. Although many abnormally spliced RNA variants and their protein products in tumor cells have been observed and their association with cancer progression was demonstrated, the biological relevance of most aberrant pre-mRNA splicing events remains unclear. Currently reported cancer-related genes with alternative splicing isoforms are involved in actually all processes generally recognized in tumorigenesis [60], including proliferation, survival, metastasis, apoptosis, angiogenesis, etc. Many alternatively spliced genes may regulate more than one of these features or signaling pathways.

For protein coding genes, splicing alterations frequently cause reading frame shift, leading to introduction of premature stop codons in mRNAs that are susceptible to NMD and thus do not produce proteins [61]. However, some aberrantly spliced transcripts, especially these without reading frame changes, can escape this surveillance mechanism and produce proteins with either defect or gain of functions. Notably, about 90% of alternative splicing events are involved in RNA sequences encoding peptide regions on protein surfaces, suggesting that alternative pre-mRNA splicing mechanism is evolutionarily selected to maximize functional diversification of the human genome [62]. ncRNA research is a very promising area with extensive relevance to cancers and an increasing number of ncRNA molecules have been identified for their regulatory functions that were previously recognized as tasks only undertaken by proteins [63]. Many ncRNAs have different alternative splicing variants and their biological significance in human diseases has not been extensively investigated [64].

## 3. Cyclin D-Binding myb-Like Transcription Factor 1 (DMTF1): A Brief Summary of Its Function

DMTF1 was identified as a CCND2 binding protein through a yeast two-hybrid screen [65]. The DMTF1 protein contains 760 amino acids and its primary structure consists of a middle DNA-binding domain and two acidic transactivation domains at the N- and C-terminals [66].

The human *DMTF1* gene is located on chromosome 7p21, a region frequently deleted in breast cancer, acute myeloid leukemia (AML), and myelodysplastic syndrome (MDS) [8,67,68,69]. *DMTF1* is highly conserved between humans and mice, with 95% similarity in amino acid sequences. Especially in the DNA binding domain of amino acids 125–417 with three myb-like repeats, human and murine DMTF1 proteins share 100% identity and the consensus binding sequence is CCCG(G/T)ATGT [70]. The predicted molecular weight of DMTF1 is 84.5 kDa, but it always migrates around 125–130 kDa in SDS-PAGE (sodium dodecyl sulfate-polyacrylamide gel electrophoresis) analyses, suggesting that the DMTF1 protein is either post-translationally modified or holding a structure unresolvable by SDS. To date, DMTF1 was only reported to be phosphorylated by CDK4 and CDK6 in presence of CCND1 [65]. Future studies are needed to determine whether other modifications contribute to the apparently slow migration of DMTF1 on SDS-PAGE and their functional relevance.

As a transcription factor, DMTF1 has been indicated to regulate multiple genes, including *ANPEP* and *CDKN2A* [66]. In addition to transcriptionally activating *CDKN2A* gene, DMTF1 directly binds MDM2 and inhibits its E3 ubiquitin ligase activity [71]. Thus, DMTF1 has the potential to positively regulate TP53 expression and cause cell cycle arrest, which has been experimentally confirmed [72]. Consistent to this regulation, *DMTF1* knockout mice exhibited compromised CDKN2A function, and *DMTF1*-null mouse embryo fibroblasts (MEFs) failed to become senescent as wild type MEFs did after 30 passages and could be morphologically transformed by oncogenic *RAS(Val12)* alone [73]. Additional studies revealed that *DMTF1*-null mice developed spontaneous malignant lymphomas and deceased from various cancers at two years of age [74]. Lymphomas could also arise from *DMTF1*(+/−) mice, suggesting that *DMTF1* is haplo-insufficient for tumor suppression. This notion is further supported by the observation that MYC-induced B-cell lymphomas dramatically reduced the time of latency at either a *DMTF1*(−/−) or *DMTF1*(+/−) genetic background. Importantly, *TP53* mutations or *CDKN2A* deletion were detected in about 50% MYC-induced B-cell lymphomas, but the concurrent *DMTF1* loss resulted in much more frequent intact *TP53* and *CDKN2A* [74]. Similarly, in both *DMTF1*(+/−) and *DMTF1*(−/−) backgrounds, the survival of *KRAS(LA)* mice was also shortened by about 15 weeks, and the lung tumors in these mice exhibited significantly reduced frequency of *TP53* mutations compared to the *DMTF1*(+/+) background [75]. In human breast cancer, *DMTF1* loss can be used to define a new disease category associated with the patient prognosis in association with CDKN2A-MDM2-TP53 pathway [76]. These data suggest that TP53 is a critical target for DMTF1 to exhibit its biological function.

DMTF1 can also activate TP53 through a CDKN2A-independent pathway. Supporting this notion, DMTF1 directly interacts with TP53, antagonizes MDM2-mediated TP53 ubiquitination and promotes TP53 nuclear localization [7]. DMTF1 increases TP53 expression and synergistically activates its target gene expression.

In addition to the regulatory role of DMTF1 in TP53 signaling pathways, DMTF1 also plays an essential role in RAS-RAF-CDKN2A signaling. In the absence of DMTF1, CDKN2A and CDKN1A activation mediated by oncogenic RAF was compromised and the cells were resistant to RAF-mediated premature senescence; thus *DMTF1*-null primary cells are susceptible to RAS-induced transformation [73].

Additional evidence for a tumor suppressive role of DMTF1 includes that the *DMTF1* promoter can be activated by oncogenes *RAS* and *HER2* but repressed by E2Fs and NFKB signals [73,77,78]. Consistently, we recently demonstrated that DMTF1α inhibits *EBRR2*-induced mammary tumorigenesis [79] and *DMTF1* loss promotes breast cancer development mediated by *CCND1* overexpression [80].

## 4. Alternative DMTF1 Pre-mRNA Splicing and Its Role in Cancer

Many splicing variants of DMTF1 pre-mRNA have been identified. In the Ensembl Project Database, 38 DMTF1 splicing variants exist and 20 of them likely encode proteins, suggesting DMTF1 pre-mRNA is differentially regulated by splicing machinery. The relative abundance and functional relevance of these transcripts and their encoded proteins need further investigation. According to the NCBI database, DMTF1 has three mRNA variants (NM_021145.3, NM_001142327.1, and NM_001142326.1) that are mostly different at the lengths of their 5′- and 3′-UTRs. Variant 1 has the longest 5′-UTR, while variant 3 possesses the longest 3′-UTR. Compared to variants 1 and 2, the 5′ side of variant 3 lacks an exon between exons 2 and 3, which consists of 117 nucleotides with the start codon ATG for variants 1 and 2. As a result, variant 3 uses a downstream ATG as a start codon, which is in frame with variants 1 and 2, and thus may produce a protein with 88 amino acids shorter at the N-terminal. However, the functional relevance of this shorter version DMTF1 remains unexplored.

**Figure 4:**
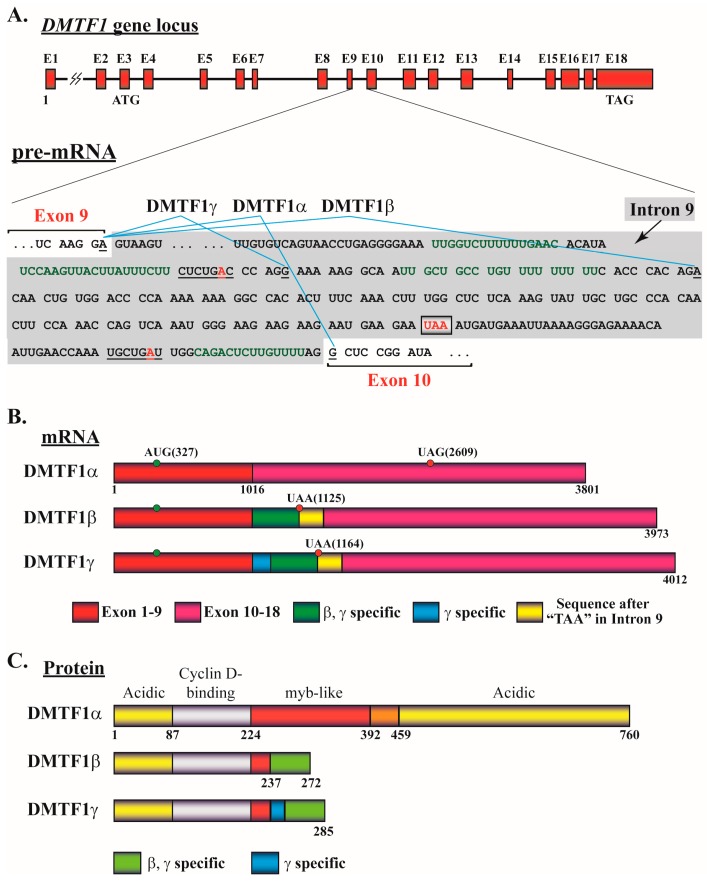
Schematic representation of the *DMTF1* gene, alternatively spliced mRNA isoforms, and proteins. (**A**) *DMTF1* gene arrangement and alternative splicing. The representation is based on the DMTF1 mRNA sequence of the accession number NM_001142327.1 in the NCBI. In the top panel, 18 exons of the *DMTF1* gene are depicted by red boxes and the introns are represented by lines. The exons and introns are drawn approximately in proportion to their relative lengths, except intron 1 (about 11 KB, the omitted region is indicated by a lightening sign). The locations of the start codon ATG (in exon 3) and stop codon TAG (in exon 18, for DMTF1α) are indicated. In the lower panel, intron 9 is presented on a gray background, while the adjacent ends of exons 9 and 10 are indicated. The last nucleotide of exon 9 (underlined, right before the donor site, GU) alternatively ligates to the three nucleotides (underlined, right after the acceptor sites, AG) in intron 9 or exon 10, which is shown by blue lines to generate DMTF1α, β, and γ isoforms. Parts of the sequence are presented as triplicates according to the reading frames of DMTF1 isoforms. The predicted consensus sites containing the “branch points” for DMTF1β/γ and DMTF1α splicing are underlined sequences CUCUGAC and UGCUGAU, respectively, with the branch points (adenosines) in red. The predicted polypyrimidine tracts for the three DMTF1 alternative splicing isoforms are in green. The stop codon TAA shared by DMTF1β and γ isoforms is in red and boxed; (**B**) Transcripts of DMTF1 isoforms. The representations for the colored box are indicated at the lower panel and the nucleotide positions of the three DMTF1 transcripts are indicated beneath them. The start codon and the stop codons (UAG and UAA) are shown for each isoform with the numbers representing their positions in mature mRNAs; (**C**) Domain structures of DMTF1 protein isoforms. The domain structures are based on a previous report [66]. The amino acid positions and lengths of the three DMTF1 protein isoforms are indicated beneath them. The representations of the colored box for β/γ- and γ-specific regions are indicated in the lower panel.

The alternative splicing between exons 9 and 10 of DMTF1 pre-mRNA was first demonstrated in 2003 by Tschan et al. [8]. In this report, two new and C-terminal-short DMTF1 isoforms, designated as DMTF1β and γ, were discovered, and the longer tumor suppressor isoform was accordingly named DMTF1α. The authors obviously used DMTF1 variant 2 (accession number NM_001142327.1), which is 3801 nucleotides in updated length and consists of 18 exons (Figure 4A,B). The two alternative splicing events of the DMTF1 pre-mRNA utilize the “intron retention” mechanism with the 3′ end of exon 9 splicing with two different sites (the 715th or 676th nucleotide of intron 9 for β and γ, respectively) in intron 9 (886 nucleotides). According to the consensus branch point-containing sequences (YNCURAY, Y: pyrimidine, R: purine, N: any nucleotide; the “A” is the branch point) [81], we identified two sites in the intron 9 as potential branch points during alternative DMTF1 pre-mRNA splicing (Figure 4A). The splicing of DMTF1β and γ likely utilizes the same branch point in a sequence CUCUGAC, while the branch point of DMTF1α splicing resides in UGCUGAU (Figure 4A). We also found that more uridines are present as potential polypyrimidine tracts upstream of the 3′ splicing sites in intron 9 for DMTF1β and γ isoforms than that of DMTF1α (Figure 4A), suggesting relatively easy spliceosome assembly for DMTF1β and γ splicing compared to DMTF1α.

The coding regions of DMTF1β and γ isoforms incidentally use an identical reading frame and thus run into the same stop codon (TAA) at the 821st nucleotide of intron 9. As a result, DMTF1β and γ proteins have primary sequences much shorter than the α (272 and 285 versus 760 amino acids). They share the first 237 amino acids with DMTF1α but suffixed by 35 and 48 amino acids, respectively, at the C-terminals that are absent in the α isoform (Figure 4C). Structurally compared to DMTF1α, DMTF1β and γ still retain the N-terminal transactivation domain (TAD) and CCND1 binding site (CBS). They only keep a small part of the myb-homology region (MHR) and lack the DNA binding ability of DMTF1α. Compared to DMTF1α, the DMTF1β transcript is highly expressed in quiescent CD34^+^ cells and peripheral blood leukocytes but shows weak expression in most other cell lines; the DMTF1γ is ubiquitously expressed at low levels [8]. Relative levels of all three proteins encoded by these DMTF1 transcripts are difficult to determine due to their limited specific regions to generate isoform-specific antibodies. We recently produced a DMTF1β-specific antibody to detect its expression in breast cancer samples [2]. Consistent with the loss of the DNA binding domain of DMTF1β and γ, neither of them could activate the *ANPEP* promoter, but DMTF1β could abrogate DMTF1α-mediated activation of the same promoter [8]. Similarly, DMTF1β and γ did not activate the *CDKN2A* promoter. However, although DMTF1γ has a very similar domain structure to DMTF1β, Tschan et al. observed that only the β, but not γ, isoform could inhibit DMTF1α-induced transactivation of the *CDKN2A* promoter in a dose-dependent manner [3]. They also indicated that DMTF1β may interact with DMTF1α to modulate its function and the ratio of DMTF1α and β are tightly regulated in hematopoietic cells. These data suggest DMTF1β’s activity in antagonizing the transcriptional activity and tumor suppressive function of DMTF1α.

Our recent study provided definitive evidence to demonstrate the oncogenic role of DMTF1β in mammary tumorigenesis, using ample data from clinical samples and transgenic mice [2]. We found that DMTF1 alternative splicing occurred in about 30% of breast cancer cases, with relatively decreased DMTF1α and increased DMTF1β expression. Consistently, our RNA-seq analyses also showed significantly increased DMTF1β transcript in 43%–55% of human breast cancer samples, different among histological subtypes. Similarly, in immunohistochemical studies, DMTF1β protein was elevated in about 60% of breast tumors compared to the surrounding normal tissues. Importantly, DMTF1 splicing favoring DMTF1β mRNA and protein overexpression was associated with poor clinical outcomes of breast cancer patients, strongly suggesting a biological function of DMTF1β during mammary tumorigenesis. In vitro experiments revealed a proliferative role of DMTF1β in mammary cells. In our in vivo studies, DMTF1β overexpression in mouse mammary driven by the *MMTV* promoter was sufficient to induce mammary gland hyperplasia and multifocal tumor lesions in mice with a mean latency of 16 months [2]. This is significantly longer than the tumor latency of *MMTV-HER2* and *MMTV-MYC* transgenic mice (about 8 and 10 months, respectively) [82,83]. On the contrary, *DMTF1α* transgenic mice displayed resistance to *HER2*-induced mammary tumor [79]. Overall, our data strongly support the notion that DMTF1 alternative splicing is a driving mechanism utilized by cancer cells to promote breast cancer development and progression. Currently, the molecular mechanisms underlying how alternative DMTF1 splicing is regulated and DMTF1β exerts its oncogenic activity still need to be explored.

## 5. Clinical Application of Alternative Splicing in Cancer Therapies

Our knowledge in understanding the mechanisms of alternative pre-mRNA splicing for cancer-related genes is important for the development of new cancer therapeutic strategies from multiple aspects, such as using cancer-specific isoforms as biomarkers and targeting oncogenic products.

### 5.1. Cancer Biomarkers

Many inherited and mutational alternative splicing mechanisms play crucial roles in human diseases, including cancers. Some alternative splicing variants are predominantly detected in tumors and thus have potential biomarker value for certain cancers [84,85]. Many cancer-related genes have been well characterized for both their functions and aberrant expression or splicing in cancers. In a study using a peptidomics approach to search for novel transcript variants in clinical proteomics, Zhang et al. identified novel alternative splicing isoform biomarkers of breast cancer [86]. In another report, Venables et al. compared alternative splicing profiles of 600 cancer-associated genes between normal and breast cancer samples, and validated 41 alternative splicing events that significantly differed among these two groups of samples. Among them, the 12 best cancer-associated splicing events can be used to identify breast cancer samples with 96% accuracy [87]. Long ncRNA MALAT1 can be alternatively spliced into two transcripts in breast cancer. The alternatively spliced shorter form has prognostic value and its expression is associated with activation of the PI3K-AKT pathway [88]. CD44 is a cell-surface receptor responsible for cell–cell communication, cell adhesion and migration, survival, and proliferation [89]. The pre-mRNA of the *CD44* gene has about 10 variable exons and thus can be theoretically spliced into up to 1000 CD44 variants (CD44v). As a transmembrane protein, the major variable region of CD44 is on the cell surface that can be heavily glycosylated. Modifications of this extracellular variable region determine its specificity as a ligand receptor. Another variable region of this protein is its cytoplasmic tail, modifications of which modulate CD44’s interaction with the cytoskeleton. Many CD44v isoforms play different roles in tumorigenesis through modulating tumor initiation or metastasis and their expression levels possess diagnostic value. For example, CD44v6, an alternative CD44 splicing variant containing exon v6, showed markedly increased levels at the late or metastatic stage of gastric and colorectal cancers [90,91], but this CD44 isoform exhibited dramatic reduction in head and neck squamous cell carcinoma [92].

The Wilms tumor 1 (*WT1*) gene encodes a zinc finger transcription factor and its inactivation is linked to Wilms tumors and some other cancers. The pre-mRNA of the *WT1* gene has 10 exons, two of which (exons 5 and 9) are alternatively spliced; exon 5 is either included or omitted, while exon 9 has two alternative splicing donor sites. Exon 5 encodes 17 amino acids serving as a binding domain for prostate apoptosis response factor 4 (PAWR, also known as PAR4) and thus the presence of exon 5 alters the cell’s response to apoptotic stimuli [93]. The alternative splicing in exon 9 of WT1 determines the inclusion of three amino acids, lysine, threonine, and serine (KTS). The presence or absence of this KTS sequence in WT1 determines its transcriptional activity, interacting proteins and subcellular localization [94]. The balance between the +KTS and -KTS isoforms correlates with the proliferation, differentiation, apoptosis, and therapeutic response of tumor cells. A study by Baudry et al. indicated that altered WT1 expression was present in 90% of Wilms tumor cases. Among them, 63% had aberrant splicing, mostly in exon 5 [95]. Many other cancer-related genes show distinct alternative splicing profiles in cancers, including *BRCA1*, *BRCA2*, *MDM2*, *KLK3* (also named prostate-specific antigen, *PSA*), and fibroblast growth factor receptors (*FGFRs*). Isoforms from the same gene always exhibit different, or even opposite, activity in modulating oncogenic signaling pathways. As indicated above, splicing factor genes, such as *SF3B1*, *SRSF2*, *U2AF1*, and *ZRSR2*, are frequently mutated in patients of MDS, although their biomarker potential and contribution to leukemia development need further investigation [20,96].

As discussed above, among the three DMTF1 isoforms, DMTF1β exhibits oncogenic activity, although the detailed mechanism of its involved signaling pathways remains to be determined. Based on the alternative splicing sites of DMTF1 pre-mRNA, we designed PCR primers to specifically detect alternative DMTF1 splicing events and generated a DMTF1β-specific antibody to analyze breast tumor samples. As a result, we observed that alternative DMTF1 splicing and DMTF1β overexpression were associated with poor clinical outcomes, suggesting a potential diagnostic value of DMTF1β for breast cancer patients [2].

### 5.2. Discovery of New Therapeutic Targets

Aberrant regulation of alternative splicing promotes cancer development and progression through generating oncogenic isoforms or reducing normal isoform expression. These also provide insights to developing new strategies in cancer therapies, such as targeting oncogenic isoforms and adjusting aberrant splicing processes.

#### 5.2.1. Targeting Oncogenic Isoforms

Aberrantly expressed protein isoforms can promote tumor development and progression. The difference in transcripts or polypeptides between defective or oncogenic variants and normal products can be used as not only cancer-associated biomarkers for diagnosis, but also susceptible targets for cancer therapies. For example, many receptors involved in cell–cell and cell–matrix interactions are mediated by alternative splicing and some of these aberrantly spliced variants can be used as biomarkers for human cancers [84,97]. Additionally, based on the difference among these variants, a straightforward strategy of inhibiting tumor cell growth is to directly target oncogenic mRNA or protein isoforms. CD44v6 is increasingly expressed in metastatic cancers. Bivatuzumab, a humanized monoclonal antibody against CD44v6, has been used in clinical trials to treat head and neck squamous cell carcinoma [98,99]. Fibronectin 1 (FN1) pre-mRNA has two alternatively spliced extracellular domains, EDA and EDB. They are large glycoproteins involved in cell adhesion and migration, and their expression is associated with a number of cancer-related biological processes. Due to specific expression in tumor cells, EDA and EDB have been extensively used in therapeutic studies for various cancers as targets of different agents, such as peptides, siRNAs, and antibodies [100,101,102,103]. The Philadelphia chromosome is a translocation between the *ABL1* gene on chromosome 9 and the *BCR* gene on chromosome 22, leading to the formation of a constitutively active hybrid tyrosine kinase that contributes to the development of leukemia, especially chronic myelogenous leukemia (CML). The alternative splicing of ABL-BCR in Philadelphia chromosome-positive leukemia produces novel tumor-specific fusion proteins that can serve as potential targets for immunotherapy of these diseases [104].

DMTF1β has a 172-nucleotide insertion in its mature mRNA and a 35-amino acid region added to its C-terminal that is not present in the tumor suppressive DMTF1α isoform [8]. These specific regions of DMTF1β can not only be used to detect this oncogenic isoform in tumor samples, but also serve as vulnerable targeting sites by therapeutic agents, such as competitive peptides, antisense oligonucleotides or siRNAs, and antibodies.

#### 5.2.2. Adjustment of Aberrant Splicing

Pharmaceutical agents can be designed to modulate aberrant splicing processes or target deregulated splicing machinery. For instance, *XBP1* plays a key role in the endoplasmic reticulum (ER) stress response and regulates the cell survival of multiple myeloma. During ER stress, the accumulation of unfolded proteins activates the inositol-requiring enzyme-1α (*ERN1*, or *IRE1α*) gene, which has RNase activity to cleave XBP1 (X-box binding protein 1) mRNA at two sites, causing unconventional alternative pre-mRNA splicing [105]. This event leads to the removal of a 26-nucleotide intron and a reading-frame shift to produce an active form of XBP1 transcription factor, which promotes the proliferation and survival of multiple myeloma cells. Ri et al. discovered that toyocamycin produced by an Actinomycete strain can specifically inhibit IRE1α-induced XBP1 alternative splicing and thus acts as a promising compound for multiple myeloma therapies [106]. In a recent report, Shkreta et al. discovered a 4-pyridinone-benzisothiazole carboxamide compound 1C8 that can modulate the splicing activity of SRSF10 and thus affect SRSF10-dependent splicing of HIV-1 [107].

Antisense oligonucleotides have been used to modulate the alternative splicing process of cancer-related genes. The underlying mechanism is to block an undesired alternative splicing by hybridizing the splice site using antisense oligonucleotides [46]. The aforementioned *BCL2L1* (or *Bcl-X*) gene encodes two alternative mRNA isoforms, Bcl-XL and Bcl-XS, with anti- or pro-apoptotic activity, respectively. As a transmembrane molecule in the mitochondria, Bcl-XL prevents CYCS (also known as cytochrome c) release and thus promotes cell survival. Bcl-XL is overexpressed in tumor cells and the ratio of Bcl-XL to Bcl-XS determines the cell fate. Taylor et al. designed antisense oligonucleotides to modulate the alternative Bcl-X pre-mRNA splicing process, leading to elevated expression of Bcl-XS and increased susceptibility of lung cancer cells to therapeutic treatment [46]. Similarly, another *BCL2* family gene, myeloid cell leukemia-1 (*MCL1*, also named *Mcl-1*), can also encode two alternatively spliced isoforms, Mcl-1L and Mcl-1S, which have anti- and pro-apoptotic functions, respectively [108]. Shieh et al. designed antisense morpholino oligonucleotides that could shift the alternative pre-mRNA splicing pattern from Mcl-1L to Mcl-1S mRNA and thus increase Mcl-1S protein expression, leading to apoptosis of skin basal cell carcinoma [109]. In another report by Giles et al., a 28-nucleotide antisense morpholino oligonucleotide to hybridize the intron 1 and translation initiation site in exon 2 of MYC pre-mRNA inhibited both alternative pre-mRNA splicing and translation of conventionally spliced MYC, and consequently induced the production of a misspliced MYC transcript and its translation [110].

A recent study by Koh et al. demonstrated that MYC regulates the core machinery for pre-mRNA splicing in lymphoma [111]. MYC upregulates the transcription of genes responsible for core small nuclear ribonucleoprotein particle assembly, maintains the splicing fidelity of specific exons, and consequently affects alternative pre-mRNA splicing, cell survival, and proliferation. A list of pre-mRNAs particularly sensitive to this regulation were identified and, importantly, antisense oligonucleotides targeting the alternatively splicing of these genes mimicked the cell-cycle arrest or apoptotic phenotypes induced by MYC depletion [111]. These data suggested the therapeutic potential of targeting aberrant splicing in cancer treatment.

We demonstrated that the ratio of alternatively spliced DMTF1β/DMTF1α isoforms was significantly increased in breast cancer samples compared to the matched normal mammary samples [2]. However, the molecular mechanisms underlying the alternative DMTF1 pre-mRNA splicing process and thus determining this ratio remain undetermined. An understanding of this splicing mechanism will help with adjusting the expression or activity of RBPs to block DMTF1β formation and increase DMTF1α expression in order to inhibit breast cancer development.

Targeting the splicing process or machinery should be done cautiously as specificity is crucial for this type of approach. This is because changes in splicing factors may affect many transcripts and thus can cause severe side effects. For example, Younis et al. identified differential multiple regulators of constitutive and alternative splicing through a reporter-based screening [112]. Some chemicals may preferentially target a family of splicing factors, but each could cause distinct splicing changes of numerous genes.

## 6. Conclusions

In the past two decades, substantial progress has been achieved in understanding the regulatory mechanisms of alternative splicing. We have demonstrated the opposite activity of DMTF1α and β isoforms in breast cancer development, and many other cancer-related genes are regulated in a similar fashion. These aberrantly spliced pre-mRNAs can be used as biomarkers for various cancers and also serve as susceptible targets in cancer therapies. The advance of our knowledge about the molecular mechanisms underlying aberrant RNA splicing in cancers will aid in designing new strategies specifically targeting oncogenic signaling in tumor cells. Overall, aberrant RNA splicing in cancers remains a fertile field to be explored and dissecting the detailed mechanisms underlying alternative pre-mRNA splicing of cancer-related genes can potentially lead to the development of novel therapeutics for cancer therapies.
